# The Potential of *N*-Acetylcysteine for Treatment of Trichotillomania, Excoriation Disorder, Onychophagia, and Onychotillomania: An Updated Literature Review

**DOI:** 10.3390/ijerph19116370

**Published:** 2022-05-24

**Authors:** Debra K. Lee, Shari R. Lipner

**Affiliations:** 1 Paul L. Foster School of Medicine, Texas Tech University Health Sciences Center El Paso, El Paso, TX 79905, USA; debra.lee@ttuhsc.edu; 2 Department of Dermatology, Weill Cornell Medicine, New York, NY 10021, USA

**Keywords:** *N*-acetylcysteine, NAC, body focused repetitive behavior, BFRB, trichotillomania, hair pulling, excoriation disorder, skin picking, onychophagia, onychotillomania

## Abstract

Background: Trichotillomania (TTM), excoriation disorder, onychophagia, and onychotillomania are categorized as body focused repetitive behavior (BFRB) disorders, causing damage to the skin, hair, and/or nails with clinically significant psychosocial consequences. Currently, there are no standardized treatments for these compulsive, self-induced disorders. Studies on treatment of these disorders using psychotropic drugs (i.e., selective serotonin reuptake inhibitors, tricyclic antidepressants, anticonvulsants) have shown variable efficacy. Recently, there is a growing interest in *N*-acetylcysteine (NAC) for treating BFRBs. NAC is a glutamate modulator that has shown promise in successfully reducing the compulsive behaviors in BFRB disorders. This article provides an updated review of the literature on the use of NAC in TTM, excoriation disorder, onychophagia, and onychotillomania. Methods: Relevant articles were searched in the PubMed/MEDLINE database. Results: Twenty-four clinical trials, retrospective cohort studies, and case reports assessing the efficacy of NAC in TTM, excoriation disorder, and onychophagia were included. No studies for onychotillomania were found in our search. Conclusions: Although NAC has proven successful for treatment of BFRB disorders, data is derived from few clinical trials and case reports assessing small numbers of patients. Larger studies with longer durations are needed to fully establish the efficacy of NAC in these disorders.

## 1. Introduction

Body-focused repetitive behavior (BFRB) disorders are self-inflicted, compulsive behaviors that cause physical damage to the skin, hair, and nails, often with psychosocial consequences. There is increased research interest in BFRBs, encompassing both psychiatry and dermatology disciplines. Approximately 30% to 40% of patients treated for dermatological conditions suffer from an underlying psychiatric disorder that worsens or causes the skin disease [1]. Common BFRBs include trichotillomania (TTM) (hair pulling), excoriation disorder (skin picking), onychophagia (nail biting), and onychotillomania (nail picking). Mild forms of these behaviors are relatively common in the general population; however, severe cases can cause significant distress or impede social functioning.

All BFRBs are now classified under obsessive compulsive and related disorders (OCRD) in the *Diagnostic and Statistical Manual of Mental Disorders, 5th edition* (DSM-5) [2]. The DSM-5 makes a distinction between BFRBs and OCRDs, stating that BFRBs are not triggered by obsessions or preoccupations, but may be preceded or accompanied by feelings of anxiety or boredom [2]. In addition, OCRD behaviors do not arise from a fixation on the body, but are provoked by other factors [2,3]. It is estimated that 1 in 20 people suffer from a BFRB [4]. The prevalence ranges from 0.5–2% for TTM [1,2,5], 1.4–5.4% for excoriation disorder [2,6], 20–30% for onychophagia [7], and 0.9% for onychotillomania [7].

Although the pathophysiology of BFRBs is incompletely understood, neuroimaging studies in patients with OCD and OCRDs have consistently shown hyperactivity in the orbitofrontal cortex and striatum [8,9]. It is hypothesized that this hyperactivity is due to an increased excitation to inhibition ratio from increased glutaminergic excitation or reduced GABAergic inhibition, resulting in the compulsive behaviors seen in BFRBs [10,11].

Effective pharmacologic treatments for BFRBs are lacking. Currently, there are no Food and Drug Administration-approved drugs for BFRBs, and psychotropic drugs with numerous side effects are often used as first-line therapy with mixed results. However, there is a growing body of evidence for the use of glutaminergic agents for treating BFRBs and OCRDs, namely *N*-acetylcysteine (NAC).

NAC (C_5_H_9_NO_3_S) is the acetylated precursor of the amino acid L-cysteine and functions as a glutamate modulator and antioxidant [12,13,14,15,16]. It is widely known as a mucolytic, an antidote for acetaminophen overdose, and a nephroprotective agent for contrast administration [15,17]. NAC attenuates glutaminergic hyperactivity by releasing glutamate, the main excitatory neurotransmitter in the central nervous system, into the extracellular space. This stimulates inhibitory glutamate receptors and reduces glutaminergic neurotransmission [1,16]. Excessive amounts of glutamate discharge results in neuronal damage and is associated with many repetitive and compulsive disorders. Significantly higher levels of glutamate have been found in the cerebral spinal fluid, orbitofrontal cortex, and caudate nucleus of OCD patients [18,19,20]. Through this mechanism of action, NAC has been successfully used as adjunctive treatment in many psychiatric mood disorders (i.e., depression, anxiety, and post-traumatic stress disorder (PTSD)) [12,21].

Abnormalities in the dopamine pathway are associated with psychiatric disorders including schizophrenia, addiction, depression, and attention deficit hyperactive disorder (ADHD). NAC indirectly regulates dopamine release through glutaminergic neurotransmission, acting on the presynaptic mGlu2/3 receptors [22]. Additionally, dopamine, glutamate, and their oxidized metabolites can be cytotoxic and contribute to oxidative stress [23,24,25]. NAC protects cells against oxidative stress by replenishing glutathione, a major antioxidant made up of glutamate, glycine, and cysteine. NAC contributes cysteine, the rate-limiting substrate of glutathione synthesis [15]. Cysteine is also an effective free radical scavenger, further minimizing inflammatory and oxidative stressors. Reduction of cellular oxidative stress is thought to block the reinstitution of compulsive behaviors [3,17].

Compared to other glutaminergic agents, NAC has received much attention for its use in treating BFRBs given its low cost and benign side effect profile. More recently, there is increasing evidence on the efficacy of NAC in treating BFRBs. This article aims to review the most recent research studies on the use of NAC in TTM, excoriation disorder, onychophagia, and onychotillomania and to assess its potential as an effective pharmacologic treatment for these disorders.

## 2. Materials and Methods

**Figure 1:**
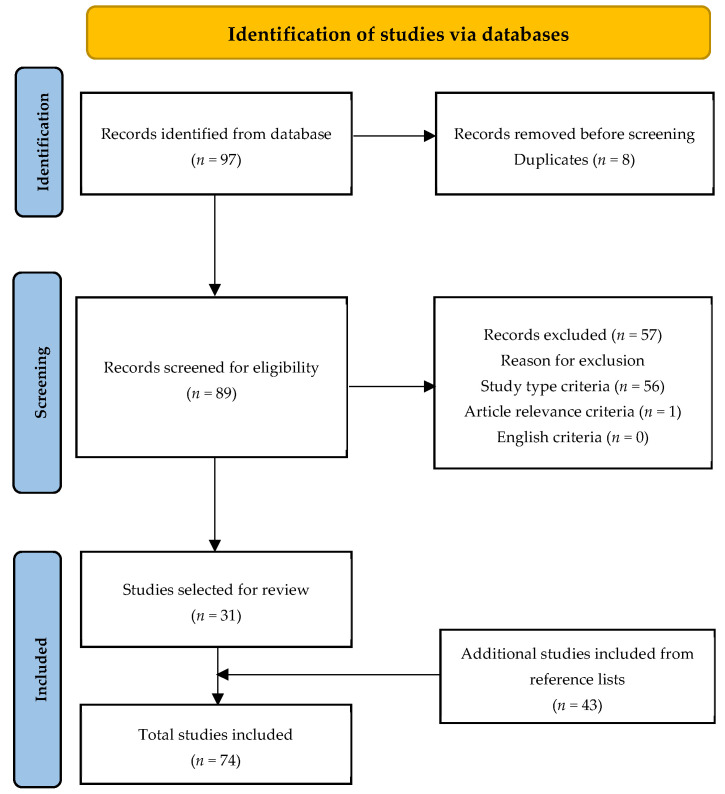
PRISMA flowchart for literature review.

We conducted literature searches for peer-reviewed articles using the PubMed/MEDLINE database in March 2022 for NAC in TTM, excoriation disorder, onychophagia, and onychotillomania using the following search terms: “N-acetylcysteine in trichotillomania”, “N-acetylcysteine in excoriation disorder”, “N-acetylcysteine in skin picking disorder”, “N-acetylcysteine in onychophagia”, and “N-acetylcysteine in onychotillomania”. Full length articles of randomized controlled trials, uncontrolled trials, systematic reviews, cross-sectional studies, cohort studies, case-controlled studies, case reports, and cases series were included. The search was limited to articles published in the English language. Reasons for exclusion are shown in the PRISMA flowchart (Figure 1). A total of 31 articles were selected for review. Reference lists in these articles were reviewed to find 43 additional articles, such that a total of 74 articles were included in this review.

## 3. NAC Considerations

NAC is inexpensive, with an average cost of 10 US dollars for one hundred 600 mg capsules, and is available as an over-the-counter supplement. NAC has a low bioavailability and can be prescribed at doses up to 2400 mg/day. NAC has also been used topically as a gel or solution for acne vulgaris and atopic dermatitis [17]. BFRBs are typically treated with oral NAC with doses ranging from 600–2400 mg/day for 1–8 months [15]. Side effects are dose dependent. Nausea, vomiting, diarrhea, and constipation have been reported with doses lower than 2400 mg/day. At doses higher than 2400 mg/day, fever, chills, skin rash, and headache may occur [3]. NAC should be used with caution in asthmatics or patients prone to anaphylactic reactions and patients susceptible to fluid overload (e.g., congestive heart failure) [26]. NAC is safe to use in pregnancy; however, it is unknown whether NAC is excreted into breast milk. NAC is completely cleared 30 h after administration. To reduce infant exposure, breast feeding women should pump and discard breast milk for 30 h after NAC administration, making NAC impractical to use in this population [26].

## 4. NAC in Trichotillomania

TTM is repetitive hair pulling of one’s own hair from the scalp, eyebrows, eyelashes, and pubic region, leading to nonscarring patchy hair loss with short hair. Patients have a negative hair pull test on physical examination. This condition is exacerbated by stress and can cause significant distress, shame, and low self-esteem. First-line pharmacologic treatments for TTM are selective serotonin reuptake inhibitors (SSRIs) and tricyclic antidepressants (TCAs), specifically clomipramine [1]. Other drugs studied for treating TTM include olanzapine, inositol, and naltrexone with limited success [1,27,28,29]. NAC has been studied in several clinical trials for TTM treatment (Table 1) [30,31,32,33,34,35,36,37,38,39,40,41].

In a 12-week randomized, double-blind, placebo-controlled trial of 50 adult patients with TTM ages 18–65 years, half of the patients received NAC (1200 mg/day), while the other half received placebo pills for six weeks. The dose was increased to 2400 mg/day in the treatment group for another six weeks, unless clinical improvement (i.e., cessation of all hair pulling) was achieved at the lower dose. Using the Massachusetts General Hospital-Hair Pulling Scale (MGH-HPS), there was a significant treatment effect after nine weeks of active medication use (*p* = 0.002) and higher efficacy in the NAC group (*F*_1,47_ = 32.152, *p* < 0.001) compared to the placebo group. Patients in the NAC group showed improvement in hair pulling severity (*F*_1,47_ = 18.245, *p* < 0.001) and resistance and control (*F*_1,47_ = 37.067, *p* < 0.001) compared to the placebo group [30].

A randomized double-blind, placebo-controlled trial of 39 pediatric TTM patients ages 8–17 years demonstrated conflicting results. Participants in the NAC group were titrated up from 600 to 2400 mg/day over four weeks, and remained on the maximum dose for the remainder of the 12-week study. The study failed to show any benefit of NAC over placebo in improving severity of TTM using the MGH-HPS (*p* = 0.55). However, all subjects, regardless of assigned group, had clinically moderate, but significant improvement in hair pulling symptoms over time (*p* = 0.002) [31]. In a follow-up longitudinal study assessing for long-term outcomes in 30 of the 39 pediatric TTM patients who stopped taking NAC, hair pulling severity on average did not differ significantly over the 3-year follow-up period (*p* = 0.77). Based on the Clinical Global Impression (CGI)-Improvement scale, 20% of patients reported very much or much improved hair pulling behavior, 40% reported no changes in their symptoms, 22% had worsened hair pulling, and 17% had significantly worsened hair pulling during the follow-up period. Subjects also reported significantly increased anxiety (*p* = 0.009) and depressive (*p* = 0.0001) symptoms at follow-up that were correlated with increased hair pulling behavior [32].

NAC has shown benefit in treating TTM in several case reports. A 40-year-old female patient with a 36-year history of TTM was successfully treated with NAC for 10 weeks. Previous treatments for her TTM included citalopram 60 mg/day, venlafaxine extended release 300 mg/day, escitalopram 30 mg/day, fluoxetine 80 mg/day, paroxetine 60 mg/day, bupropion sustained release 300 mg/day, clomipramine 150 mg/day, lithium 900 mg/day, and olanzapine 10 mg/day, with each medication trial lasting at least 16 weeks. Additionally, the patient received cognitive behavioral therapy (CBT) and habit reversal therapy (HRT) for 12 weeks without any decrease in her hair pulling symptoms. The patient was started on NAC 600 mg/day and gradually increased to 1800 mg/day. After 4 weeks, her hair pulling urges decreased. After another dose increase to 2400 mg/day for 2 weeks, the patient noted compete cessation of hair pulling behavior with maintenance of results after 5 months [41].

In another case, a 58-year-old female with TTM was successfully treated with NAC 1200 mg/day for 32 weeks with complete and sustained recovery. After 4 weeks on NAC, the patient noticed modest regrowth of her scalp, which further improved at 10 weeks. The patient remained on the dose for a total of 32 weeks. This is the longest reported treatment duration for any BFRB with NAC. The patient also did not report any adverse side effects during the entire treatment duration [39].

Of note, some cases reported combined treatments of NAC with other psychotropic medications, making it unclear whether NAC alone would produce similar results. For example, a 25-year-old female with TTM comorbid with binge eating disorder, depression, and anxiety was treated with NAC starting at 600 mg/day and titrated up to 1800 mg/day, fluvoxamine 150 mg/day, and bupropion 300 mg/day with significantly reduced hair pulling behavior and binge eating urges. After two weeks of treatment, the patient had almost no hair pulling and noted hair thickening. At 14 weeks, the patient reported no hair pulling behavior or binge eating episodes with improved anxiety and depression symptoms [33].

A 14-year-old girl with TTM comorbid with ADHD saw significant improvement of hair pulling behavior after 2 weeks of treatment with NAC 1200 mg/day. Before starting NAC, the patient was taking haloperidol and methylphenidate for her ADHD for 6 months and 3 years, respectively. Upon starting NAC, haloperidol was discontinued. There was significant improvement in hair pulling after 2 weeks and complete hair growth was noted after 6 months [38].

In another case, an 18-year-old female was treated with NAC 2700 mg/day, fluoxetine 40 mg/day, and psychotherapy for TTM comorbid with excoriation disorder, OCD, depression, and anxiety. After 16 weeks of treatment, the patient reported significant reduction of her hair pulling and skin picking behavior and decreased severity of her comorbid psychiatric symptoms. Although the patient did not achieve complete remission, she was satisfied with the improvement in symptoms. This is the highest reported dosage for NAC in hair pulling, as 2400 mg/day is usually the maximum dose prescribed. The patient did not report any adverse effects despite the high dosage [34].

## 5. NAC in Excoriation Disorder

Excoriation disorder, also known as skin picking disorder, refers to compulsive pathological skin picking, leading to skin damage and scarring. This condition can be triggered by stress, anxiety, and sensation or appearance of a bump, blemish, or discoloration of the skin [17]. In severe cases, tissue damage as a result of self-inflicted picking can lead to localized and systemic infections. Behavioral therapy, including CBT and HRT, is a non-pharmacological treatment for excoriation disorder [42,43,44]. SSRIs, such as fluoxetine, citalopram, fluvoxamine, and sertraline, are the most studied drugs for the treatment of skin picking disorder. Other pharmacologic treatments include lamotrigine, atomoxetine, methylphenidate, and inositol [42,45]. In recent years, studies exploring the efficacy of NAC in excoriation disorder have revealed promising results (Table 2) [35,41,46,47,48,49,50,51,52].

In a 12-week randomized, double-blind trial of 66 adult patients, of which half were treated with NAC (1200 mg/day titrated up to 3000 mg/day) and half with placebo, the NAC group had significant improvements in skin picking severity using a modified Yale-Brown Obsessive Compulsive Scale (NE-YBOCS) (*p* = 0.048). The CGI-Severity scale was used as a secondary efficacy measure of skin picking severity and also showed improved symptom severity at the end of the 12-week trial compared to baseline scores (*p* = 0.03) [46]. The high NAC dose was well tolerated among patients in the treatment group and the only adverse events reported were nausea (*n* = 5), dry mouth (*n* = 1), constipation (*n* = 2), and dizziness (*n* = 1). No patients were withdrawn from the study due to these effects. Another retrospective cohort study of 28 adult patients with skin picking treated with NAC was performed to corroborate the results of the previous randomized controlled trial. Patients were given a dose range of 1200–2400 mg/day for 12 consecutive weeks. There were no defined outcome measures. Patients were judged as having a positive treatment response if any objective improvement was documented in the physical examination and assessment sections of the patient’s note. Of the 13 patients that completed the trial, 61.5% showed a positive response. The study also reported that 40% of patients terminated treatment before the recommended trial period due to lack of improvement of skin picking behavior [47].

An open-label pilot study assessed the efficacy of NAC in treating skin picking disorder in 35 pediatric and adult patients with Prader—Willi syndrome. The study found reduced skin picking behaviors in all patients after 12 weeks of NAC treatment (450–1200 mg/day) and 71% of patients reported complete resolution, which was defined as completely healed or scarred over lesions [48]. Only two patients experienced side effects from NAC, which were mild and included gastrointestinal cramping, flatulence, and diarrhea. These effects improved after a few weeks of treatment. However, conflicting results were shown in a retrospective study of 14 German adult skin pickers with Prader—Willi syndrome treated with NAC 1800–2400 mg/day for 12 weeks. Based on the CGI-Improvement scale, six patients had improved skin picking symptoms, six patients had no improvement, and two patients had worsening symptoms, suggesting limited efficacy for NAC in excoriation disorder [49].

Several case reports have shown treatment success for NAC in excoriation disorder. A 12-year-old female patient achieved complete remission of skin picking behavior with NAC after four years of treatment failure with habit reversal therapy, fluoxetine, sertraline, olanzapine, aripiprazole, and valproic acid. After only two weeks of NAC 600 mg/day, the patient reported fewer urges to pick with a CGI-Improvement scale score of 3 or “Minimally Improved”. The dose was increased to 1200 mg/day for four weeks and skin picking impulses were further reduced. After a final dose increase to 1800 mg/day for four weeks, no new lesions were noted with a CGI-Improvement scale score of 1 or “Very Much Improved”, denoting complete cessation of skin picking [52].

A 52-year-old female patient with excoriation disorder, comorbid with bulimia nervosa and compulsive shopping, had rapid improvement of skin picking behavior while on NAC 1200 mg/day. The patient was treated with 600 mg/day for one week, after which the dose was increased to 1200 mg/day for three weeks. Within one week of the new dose, the patient had a reduction in her skin picking urges and behavior by 50%. The dose was increased to 1800 mg/day and the patient reported complete absence of picking and occasional urges within two days of starting the new dose. The patient maintained these results without any adverse side effects for another four months, while continuing the 1800 mg/day dosage [41].

A 42-year-old female with excoriation disorder comorbid with major depression saw improvements in her skin picking behavior with concurrent treatment of NAC 1200 mg/day and venlafaxine 225 mg/day. After a venlafaxine dose increase from 75 mg to 225 mg daily and only partial relief of her skin picking and depressive symptoms, NAC was added. Ten days after starting NAC, the patient reported a decrease in skin picking. At her next follow-up visit, the patient reported a 70% decrease in her skin picking symptoms [35].

## 6. NAC in Onychophagia and Onychotillomania

Onychophagia refers to chronic biting of the nail plate, nail folds, and/or cuticle and onychotillomania is defined as repetitive nail picking or pulling. Both conditions can lead to damage of the nail unit and periungual skin. Nail biting and nail picking have been less frequently studied compared to TTM and excoriation disorder. Behavior modification therapies, including CBT and HRT, are considered first-line treatments for onychophagia and onychotillomania. Pharmacological treatments include SSRIs, TCAs, bupropion, and lithium for onychophagia, and SSRIs, TCAs, thioridazine, pimozide, and triamcinolone acetonide injections for onychotillomania [53,54,55,56,57]. NAC has been studied for onychophagia, but not for onychotillomania (Table 3) [35,58,59,60]. However, the efficacy of NAC in treating onychophagia and other BFRBs suggests potential treatment success in onychotillomania.

A 12-week randomized, double-blind, placebo-controlled clinical trial studied NAC in 42 children and adolescent nail biters ages 6 to 18 years. Patients in the treatment group were given NAC 800 mg/day. The study reported significantly different nail lengths between the treatment group (5.21 mm) and placebo (1.18 mm) after the first month of treatment (*p* < 0.04). However, there was no significant difference after two months of treatment. Side effects reported in the treatment group included headache, agitation, and social withdrawal in one patient, while another patient reported severe aggression, both resulting in study withdrawal [58]. These results suggest that NAC may decrease nail biting behavior in the short-term. However, a low dosage of NAC was used in the study, possibly contributing to the reduced treatment effect.

A 24-year-old male nail biter who also consumed his nails after biting was successfully treated with NAC 1200–1800 mg/day for six weeks. He was previously on a one-month trial of paroxetine four years prior without any improvements in his nail biting behavior. After three weeks on the 1800 mg/day dosage, the patient lost his urge to bite his nails and even used nail clippers for the first time. After six weeks of treatment, nail biting completely ceased without any new symptoms at follow-up visits [35].

NAC has also been shown to reduce nail biting behaviors in adult patients with comorbid psychiatric conditions. For example, in one case report of an autistic 8-year-old boy with severe onychophagia, NAC 800 mg/day was used to successfully treat his nail biting. For two years, the patient was treated with risperidone 2 mg/day and thioridazine 10 mg/day to control his hyperactivity and inattentiveness. NAC was added for management of his nail biting, and after one month of treatment, the patient’s parents noticed marked reduction in both nail biting and autism symptoms [59].

A female 46-year-old lifelong nail biter with bipolar disorder treated with lithium 900 mg and quetiapine 300 mg daily saw significant improvement in nail biting symptoms with NAC 2000 mg/day. After two weeks of treatment, the patient completely stopped biting her nails and results were maintained at her seven-month follow-up. She also reported stronger nails compared to strength prior to NAC treatment [60].

A 44-year-old female with onychophagia for her whole life, depression, and anxiety was successfully treated for her nail biting with NAC 2000 mg/day. The patient was also treated with mirtazapine 15 mg daily for her comorbid psychiatric conditions. After four months of NAC treatment, the patient ceased all nail biting for the first time in ten years. Two months later, efficacy was maintained [60].

Currently, no studies have been performed investigating efficacy of NAC in treating onychotillomania alone. However, a group of investigators reported on the use of NAC 1200–2400 mg/day to successfully treat several patients with both chronic nail picking and nail biting [61]. Given the similar pathophysiology and overlap of onychotillomania with onychophagia, TTM, and excoriation disorder, NAC may have comparable results to other BFRBs.

## 7. Discussion

Due to the lack of efficacious pharmacologic agents with minimal side effects for the treatment of BFRBs, there is a need for alternative therapies. The glutaminergic modulator and antioxidant NAC has received much attention, and there have been a number of studies assessing its efficacy for BFRBs. While there is sufficient preclinical evidence and theoretical justification for NAC in treating BFRBs, there is still a paucity of long-term clinical trials evaluating large sample sizes. Additionally, there are several unknowns in using NAC for BFRB treatment.

The effective dose of NAC is uncertain as many studies use various dosages, especially in pediatric vs. adult populations. It is possible that some studies used a subtherapeutic dose. In Ghanizadeh et al.’s pediatric onychophagia trial, the maximum NAC dose administered was 800 mg/day, which is substantially lower compared to other pediatric trials (e.g., 2400 mg/day) [58]. Using a higher dose may have impacted the results of the study. Additionally, most clinical trial durations were short, with most lasting only three months. There is evidence in longer clinical trials (e.g., 16 and 20 weeks) investigating NAC for OCD that do not show significant improvement of symptoms by the study endpoint based on YBOCS scores [62,63,64]. Perhaps more clinical trials with longer durations are necessary to fully assess whether NAC remains efficacious in the long-term term treatment of BFRBs.

Several factors may have led to an overestimation of the effect of NAC in the mentioned studies. Concurrent treatments reported in some studies, both pharmacological and non-pharmacological, make it unclear whether NAC alone would produce the same results. For example, one patient with TTM and several comorbid psychiatric conditions was treated with psychotherapy, fluoxetine, and NAC. Although fluoxetine was prescribed to target comorbidities, it may have positively influenced the treatment of TTM [34]. Similarly, in another patient treated for TTM, NAC was added only after starting citalopram for several weeks when the SSRI could have elicited improvements in hair pulling [35].

Psychiatric comorbidities were reported in most studies. The presence of these conditions may lessen the therapeutic effects of NAC in treating BFRBs. This was likely the case in the same patient from Jones et al. with TTM comorbid with excoriation disorder, OCD, depression, and anxiety. Despite a high NAC dose of 2700 mg/day, the patient was unable to reach full remission for her TTM [34]. Moreover, adult patients generally have longer disease duration with more comorbidities compared to children. These factors should be considered in future studies when investigating adult and pediatric patients.

Another challenge in studying NAC is medication adherence. Although most clinical trials were in controlled settings, the studies mostly occurred outpatient without strong emphases on adherence. Given NAC’s poor bioavailability, poor medication compliance impacts the efficacy of the drug and may explain some mixed results [65].

## 8. Limitations and Future Directions

Future clinical trials are needed to address methodological concerns and better determine the benefits of NAC for BFRBs. Larger sample sizes with longer treatment durations and follow-up times are necessary to assess for maintenance of treatment results. Further, studies for determining the most effective dosage of NAC are also important, especially in pediatric vs. adult patients, as it remains uncertain if higher doses correlate with greater efficacy.

NAC usefulness may also be limited due to its low bioavailability. There is evidence that esterification of the carboxyl group in NAC to produce *N*-acetylcysteine ethyl ester (NACET) increases the lipophilicity of the compound, resulting in improved pharmacokinetics [66]. NACET could be utilized in future clinical trials as it is potentially more effective than NAC.

Other pharmacological agents such as memantine and glycine have been studied for refractory OCD with some success and could be applied for treatment of BFRBs [67,68,69]. These alternative glutaminergic agents work similarly to NAC and exert their effects on glutamate via the NMDA receptor. So far, these drugs have only been studied as adjunctive treatment in OCD and more research is needed to determine their effect in treating BFRBs. Non-pharmacological therapies that can be explored for the treatment of BFRBs include non-invasive brain stimulation (NIBS) techniques. Similar to NAC, NIBS is well-tolerated, economical, and convenient. NIBS has been previously studied in other psychiatric conditions and functions by passing an electrical current through the cerebral cortex. This current penetrates the scalp and modulates cortical excitability [70]. NIBS has been successful in targeting the aberrant amygdala-medial prefrontal cortex-hippocampus neural circuit associated with abnormal fear memory acquisition and consolidation to treat specific phobias and other mood disorders such as PTSD, depression, and anxiety [70,71]. In refractory cases that do not respond to psychotherapy and pharmacological therapies, NIBS has also been shown to be an efficacious alternative treatment [72]. Moreover, there is research to support NIBS in the treatment of OCD and its use in conjunction with psychotherapy [73,74]. Taken together, there is great potential in applying NIBS to the treatment of BFRBs.

## 9. Conclusions

NAC has shown promise in treating BFRBs compared to other previously studied drugs, due to efficacy, low side effect profile, and affordability. Based on our literature review, there are only two clinical trials for TTM, one for excoriation disorder, one for onychophagia, and none for onychotillomania. Therefore, there is a need for more research in use of NAC for BFRB treatment. Until more clinical trials with larger sample sizes and longer durations for adult and pediatric populations are available, there is currently not enough evidence to firmly recommend NAC for TTM, excoriation disorder, onychophagia, or onychotillomania.

## Figures and Tables

**Table 1:** Summarizes all NAC treatment studies for TTM, which includes one adult and one pediatric randomized double-blind controlled trials and nine case reports.

Summary of NAC Treatment Studies in Trichotillomania							
Study	Design	Patients	Age (Year)	Comorbidities	NAC Dose	Other Concurrent Medications	Outcomes
Grant, Odlaug, and Kim (2009) [30]	RDBPCT	Adult (*n* = 50)	18–65	Depression, anxiety, OCD, PTSD, SPD, bulimia	1200–2400 mg/day	SSRIs, SNRIs, stimulants, psychotherapy	The NAC group showed higher efficacy (*F*_1,47_ = 32.152, *p* < 0.001) compared to the placebo group based on MGH-HPS. The NAC group also showed improvement in hair pulling severity (*F*_1,47_ = 18.245, *p* < 0.001) and resistance and control (*F*_1,47_ = 37.067, *p* < 0.001) compared to placebo.
Bloch et al. (2013) [31]	RDBPCT	Pediatric (*n* = 39)	8–17	ADHD, depression, anxiety, OCD, tic disorder, SPD	600–2400 mg/day	SSRIs, antipsychotics, atomoxetine, psychotherapy	No significant difference between NAC and placebo group based on MGH-HPS (*p* = 0.55). Moderate decrease in hair pulling noted in both groups (*p* = 0.002).
Zhao et al. (2021) [33]	Case report	Adult (*n* = 1)	25, F	BED, anxiety, depression	600–1800 mg/day	Fluvoxamine 150 mg/day, bupropion 300 mg/day	After 2 weeks, stable mood and reduced hair pulling behavior reported. At 14 weeks, patient reported no hair pulling or binge eating episodes, and improved anxiety and depression.
Jones, Keuthen, and Greenberg (2018) [34]	Case report	Adult (*n* = 1)	18, F	OCD, depression, anxiety, SPD	2700 mg/day	Fluoxetine 40 mg/day, psychotherapy	After 16 weeks, patient had significant reduction in hair pulling, skin picking, depression, anxiety, and OCD symptoms. Full remission was not reached.
Kilic and Keles (2018) [35]	Case report	Adult (*n* = 1)	18, F	Depression, anxiety	1200 mg/day	Fluoxetine 40 mg/day	After 3 weeks, patient showed decreased hair pulling urges and behavior. All depression and anxiety symptoms ceased. At 6-month follow-up, no hair pulling was noted.
Pino et al. (2017) [36]	Case report	Pediatric (*n* = 1)	12, F	Not specified	2400 mg/day	Doxepin 10 mg/day, fluoxetine 20 mg/day, pimozide 2 mg/day	After 6 months, patient had improved hair density and dermoscopy findings.
Barroso et al. (2017) [37]	Case report	Pediatric (*n* = 1)	11, M	Asthma, atopic dermatitis	1200–1800 mg/day	None	After 3 months, patient showed improvement at 1200 mg/day. Remission with complete hair regrowth was achieved at 1800 mg/day for 3 months.
Ozcan and Seckin (2016) [38]	Case report	Adult and pediatric (*n* = 2)	30, F; 14, F	Not specified; ADHD	1200 mg/day	None; methylphenidate	Case 1: After 2 months, hair pulling decreased with complete remission within 4 months. No recurrence of hair pulling was noted at 7-month follow-up. Case 2: After 2 weeks, significant improvement of hair pulling noted with complete hair regrowth after 6 months. No recurrence of hair pulling noted at 8-month follow-up.
Taylor and Bhagwandas (2014) [39]	Case report	Adult (*n* = 1)	58, F	Unexplained weight loss	1200 mg/day	None	After 4 weeks, patient showed noticeable regrowth of hair, which further improved at 10 weeks. Progress continued and maintained at 32 weeks.
Rodrigues-Barata et al. (2012) [40]	Case report	Adult (*n* = 2)	23, F; 19, F	Alopecia; Not specified	1200 mg/day	None	Case 1: Within 2 months, hair regrowth was observed; Case 2: Complete regrowth was observed after 3 months of treatment.
Odlaug and Grant (2007) [41]	Case report	Adult (*n* = 2)	28, M; 40, F	ADHD, nail biting; Not specified	600–1800 mg/day; 600–2400 mg/day	None	Case 1: Dose was increased from 600 to 1800 mg/day over several weeks. Complete cessation of hair pulling after 1 week on 1800 mg/day. Case 2: Dose was increased from 600 to 2400 mg/day. Complete cessation of urges and hair pulling after 2 weeks on 2400 mg/day.

NAC, N-acetylcysteine; RDBPCT, randomized double blind placebo controlled trial; ADHD, attention deficit hyperactive disorder; OCD, obsessive-compulsive disorder; SPD, skin picking disorder; TTM, trichotillomania; PTSD, post-traumatic stress disorder; BED, binge eating disorder; SSRIs, selective serotonin reuptake inhibitors; SNRIs, serotonin-norepinephrine reuptake inhibitors; TCAs, tricyclic antidepressants; MGH-HPS, Massachusetts General Hospital-Hair Pulling Scale; NE-YBOCS, Yale-Brown Obsessive Compulsive Scale; CGI, Clinical Global Impression.

**Table 2:** Summarizes all NAC treatment studies for excoriation disorder, which includes one adult randomized double-blind controlled trial, two adult retrospective cohort studies, one adult and pediatric open-label pilot study, and five case reports.

Summary of NAC Treatment Studies in Excoriation Disorder.							
Study	Design	Patients	Age (Year)	Comorbidities	NAC Dose	Other Concurrent Medications	Outcomes
Grant et al. (2016) [46]	RDBPCT	Adult (*n* = 66)	18–65	Depression, anxiety, TTM, nail biting	1200–3000 mg/day	Psychotropic medications	After 12 weeks, NAC treatment group showed significant improvement in skin picking severity compared to placebo based on NE-YBOCS and CGI-Serverity scale (*p* = 0.048, *p* = 0.03).
Hwang, Campbell, and Sartori-Valinotti (2021) [47]	Retrospective cohort study	Adult (*n =* 28)	Mean: 57.2	Not specified	1200–2400 mg/day	Doxepin, duloxetine	After 12 weeks, 61.5% of patients reported a positive response to NAC.
Miller and Angulo (2013) [48]	Open-label pilot study	Adult and pediatric (*n* = 35)	5–39	Prader—Willi syndrome	450–1200 mg/day	Valproic acid, quetiapine, risperidone, spironolactone, growth hormone, metformin, levothyroxine, modafinil	After 12 weeks, 100% of patients reported reduced skin picking behavior and 71% achieved complete resolution.
Wieting et al. (2021) [49]	Retrospective cohort study	Adult (*n* = 14)	17–53	Prader—Willi syndrome	1800–2400 mg/day	Risperidone, pipamperone, aripiprazole, sertraline, milnacipran	After 12 weeks, 6 patients reported no changes in symptoms and 2 had worsened symptoms based on CGI-Improvement scale.
Ozcan (2021) [50]	Case report	Adult (*n* = 2)	75, F; 36, F	Not specified	1200 mg/day	None	Case 1: After 2 weeks, patient reported decreased skin picking. Treatment was continued for 3 months and no relapse at 6-month follow-up. Case 2: After 6 weeks, complete cessation skin picking reported and no relapse at 3-month follow-up.
Kilic and Keles (2019) [35]	Case report	Adult (*n* = 1)	42, F	Depression	1200 mg/day	Venlafaxine 225 mg/day	After 10 days, patient reported decreased skin picking symptoms. Complete cessation of skin picking achieved at 3 months.
Silva-Netto et al. (2014) [51]	Case report	Adult (*n* = 3)	45, F; 40, F; 31, F	TTM, depression; Bipolar disorder; Depression, pathological jealousy, internet addiction	1200–1800 mg/day; 1200 mg/day; 1200 mg/day	Venlafaxine 75 mg/day; lithium 600 mg/day, quetiapine 50 mg/day; fluoxetine 20 mg/day	Case 1: Skin picking resolved completely. Case 2: Complete resolution of skin picking achieved and maintained for 10 months. Case 3: Substantial improvement of skin picking.
Percinel and Yazici (2014) [52]	Case report	Pediatric (*n* = 1)	12, F	Not specified	600–1800 mg/day	None	4 weeks after a dose increase from 600 to 1200 mg/day, skin picking urges and behavior decreased dramatically and complete remission achieved after 4 weeks on 1800 mg/day.
Odlaug and Grant (2007) [41]	Case report	Adult (*n* = 1)	52, F	Bulimia nervosa, compulsive buying	600–1800 mg/day	None	Patient reported 50% decrease of picking urges and behaviors within 1 week after dose increase from 600 to 1200 mg/day. Patient had no skin picking behavior after a dose increase to 1800 mg/day.

NAC, N-acetylcysteine; RDBPCT, randomized double blind placebo controlled trial; ADHD, attention deficit hyperactive disorder; OCD, obsessive-compulsive disorder; SPD, skin picking disorder; TTM, trichotillomania; PTSD, post-traumatic stress disorder; BED, binge eating disorder; SSRIs, selective serotonin reuptake inhibitors; SNRIs, serotonin-norepinephrine reuptake inhibitors; TCAs, tricyclic antidepressants; MGH-HPS, Massachusetts General Hospital-Hair Pulling Scale; NE-YBOCS, Yale-Brown Obsessive Compulsive Scale; CGI, Clinical Global Impression.

**Table 3:** Summarizes all NAC treatment studies for onychophagia, which includes one pediatric randomized double-blind controlled trials and three case reports.

Summary of NAC Treatment Studies in Onychophagia							
Study	Design	Patients	Age (Year)	Comorbidities	NAC Dose	Other Concurrent Medications	Outcomes
Ghanizadeh et al. (2013) [58]	RDBPCT	Pediatric (*n* = 42)	6–18	OCD, depression, anxiety, ADHD, tic disorder, SPD	200–800 mg/day	SSRIs, SNRIs, stimulants, antipsychotics, TCAs	Significantly increased nail length in treatment group compared to placebo after 1 month (*p* < 0.04). No significant difference noted after 2 months.
Kilic and Keles (2019) [35]	Case report	Adult (*n* = 1)	24, M	Not specified	1200–1800 mg/day	None	After 3 weeks on 1800 mg/day, patient lost urge to bite his nails. Efficacy was maintained after 6 weeks.
Ghanizadeh and Derakhshan (2012) [59]	Case report	Pediatric (*n* = 1)	8, M	Autism	800 mg/day	Risperidone 2 mg/day and thioridazine 10 mg/day	After 1 month of NAC, patient’s parents reported reduced nail biting and autism symptoms.
Berk et al. (2009) [60]	Case report	Adult (*n* = 3)	46, F; 44, F; 46, M	Bipolar disorder; Depression, anxiety, bipolar disorder; Depression, bipolar disorder	2000 mg/day; 2000 mg/day; Not specified	Lithium 900 mg/day, quetiapine 300 mg/day; Mirtazapine 15 mg; None	Case 1: After 2 weeks, patient completely stopped nail biting and results were maintained at 7-month follow-up; Case 2: After 4 months, patient completed stopped nail biting and results were maintained at 2-month follow-up; Case 3: After 28 weeks, patient reported reduction in nail biting.

NAC, N-acetylcysteine; RDBPCT, randomized double blind placebo controlled trial; ADHD, attention deficit hyperactive disorder; OCD, obsessive-compulsive disorder; SPD, skin picking disorder; TTM, trichotillomania; PTSD, post-traumatic stress disorder; BED, binge eating disorder; SSRIs, selective serotonin reuptake inhibitors; SNRIs, serotonin-norepinephrine reuptake inhibitors; TCAs, tricyclic antidepressants; MGH-HPS, Massachusetts General Hospital-Hair Pulling Scale; NE-YBOCS, Yale-Brown Obsessive Compulsive Scale; CGI, Clinical Global Impression.

## Data Availability

Not applicable.
